# A global time series of traffic volumes on extra-urban roads

**DOI:** 10.1038/s41597-024-03287-z

**Published:** 2024-05-08

**Authors:** Maarten J. van Strien, Adrienne Grêt-Regamey

**Affiliations:** Planning of Landscape and Urban Systems PLUS, ETH Zurich, Stefano-Franscini-Platz 5, CH-8093 Zurich, Switzerland

**Keywords:** Environmental impact, Biodiversity, Ecosystem ecology

## Abstract

Traffic on roads outside of urban areas (i.e. extra-urban roads) can have major ecological and environmental impacts on agricultural, forested, and natural areas. Yet, data on extra-urban traffic volumes is lacking in many regions. To address this data gap, we produced a global time-series of traffic volumes (Annual Average Daily Traffic; AADT) on all extra-urban highways, primary roads, and secondary roads for the years 1975, 1990, 2000 and 2015. We constructed time series of road networks from existing global datasets on roads, population density, and socio-economic indicators, and combined these with a large collection of empirical AADT data from all continents except Antarctica. We used quantile regression forests to predict the median and 5% and 95% prediction intervals of AADT on each road section. The validation accuracy of the model was high (pseudo-R^2^ = 0.7407) and AADT predictions from 1975 were also accurate. The resulting map series provides standardised and fine-scaled information on the development of extra-urban road traffic and has a wide variety of practical and scientific applications.

## Background & Summary

Globally, road transport has increased strongly in the past decades and this trend is expected to continue in the coming decades^1^. Although roads and mobility generate socio-economic benefits^2^, roads and traffic also have numerous negative impacts on the environment^3^, ecology^4,5^, climate^6^ and human health and well-being^7,8^. Although the presence or absence of a road is undoubtedly the ultimate cause of such negative impacts, the traffic volume is usually the main proximate driver of the magnitude of these effects^9–11^. For instance, Kim, *et al*.^12^ found a strong positive correlation between traffic volumes and concentrations of certain persistent organic pollutants in roadside soils. Similarly, the incidence of asthma cases was positively related to proximate traffic volumes^13^. Also ecological studies have found that the severity of road-related threats to wildlife populations is strongly dependent on traffic volumes^10^. These examples underline the importance of traffic volume data to locate areas where the detrimental impacts of roads are highest and to plan mitigation strategies. A careful planning of roads can strongly reduce their negative impacts^14^. Despite the importance of traffic volume data, in many countries such data is either lacking^15–17^ or efforts to establish such datasets are mainly focussed on urban areas e.g.^18–23^. This fragmentation of traffic data within and between countries poses challenges in comparing traffic trends across different regions and over time.

Although traffic outside of urban areas (i.e. extra-urban traffic) thus receives relatively little attention, its ecological and environmental impacts are not less severe than those of urban traffic. Extra-urban roads usually account for the majority of the total road length in a country^24,25^. Compared to urban trips, extra-urban trips usually have a low frequency^26^, but a longer length, and therefore account for a considerable proportion of the total distance travelled by car^25,27^. As extra-urban roads intersect the landscape far beyond the boundaries of cities traversing rural and more natural landscapes^24,28^, extra-urban traffic can have a considerable impact on the ecology and environment of these places. Extra-urban traffic can deteriorate agricultural production, endanger the functioning of ecosystems through nitrogen deposition^29^ or other forms of noise, water and soil pollution^30–32^ and jeopardise species survival by degrading and fragmenting habitats^33^. As the amount of traffic on extra-urban roads is largely driven by demographic and socio-economic conditions in urban regions^26^, extra-urban traffic thus forms an important telecoupling between populated and depopulated places. To quantify and mitigate the negative impacts of extra-urban traffic, there is an urgent need for harmonised, large-scale traffic volume estimates on extra-urban roads.

Many studies have focussed on developing models to extrapolate and predict traffic volumes across a road network^16,34–37^. Particularly on the vast stretches of rural and low-volume roads, such methods provide a fast and low-cost alternative to laborious and expensive empirical measurements of traffic volume^16,34,35^. Furthermore, traffic prediction models allow to create traffic datasets that are comparable across space and time. A commonly used metric for traffic volume in these studies is the Annual Average Daily Traffic (AADT), which is “the average number of vehicles that pass a roadway section each day in a particular year”^36^^, p. 2979^. A variety of regression techniques has been used to model AADT ranging from traditional linear regression^38^ to contemporary machine learning approaches^16^. In recent years, graph theoretical approaches have also been added to the suite of methods to model AADT e.g.^37^. These models can not only be used to predict AADT on road sections within a current road network, but also to make predictions of AADT for past or future years^36^. Despite the advances made with the modelling and predicting of AADT across road networks, none of the methods has been applied at a global scale, let alone for multiple years.

**Fig. 1:**
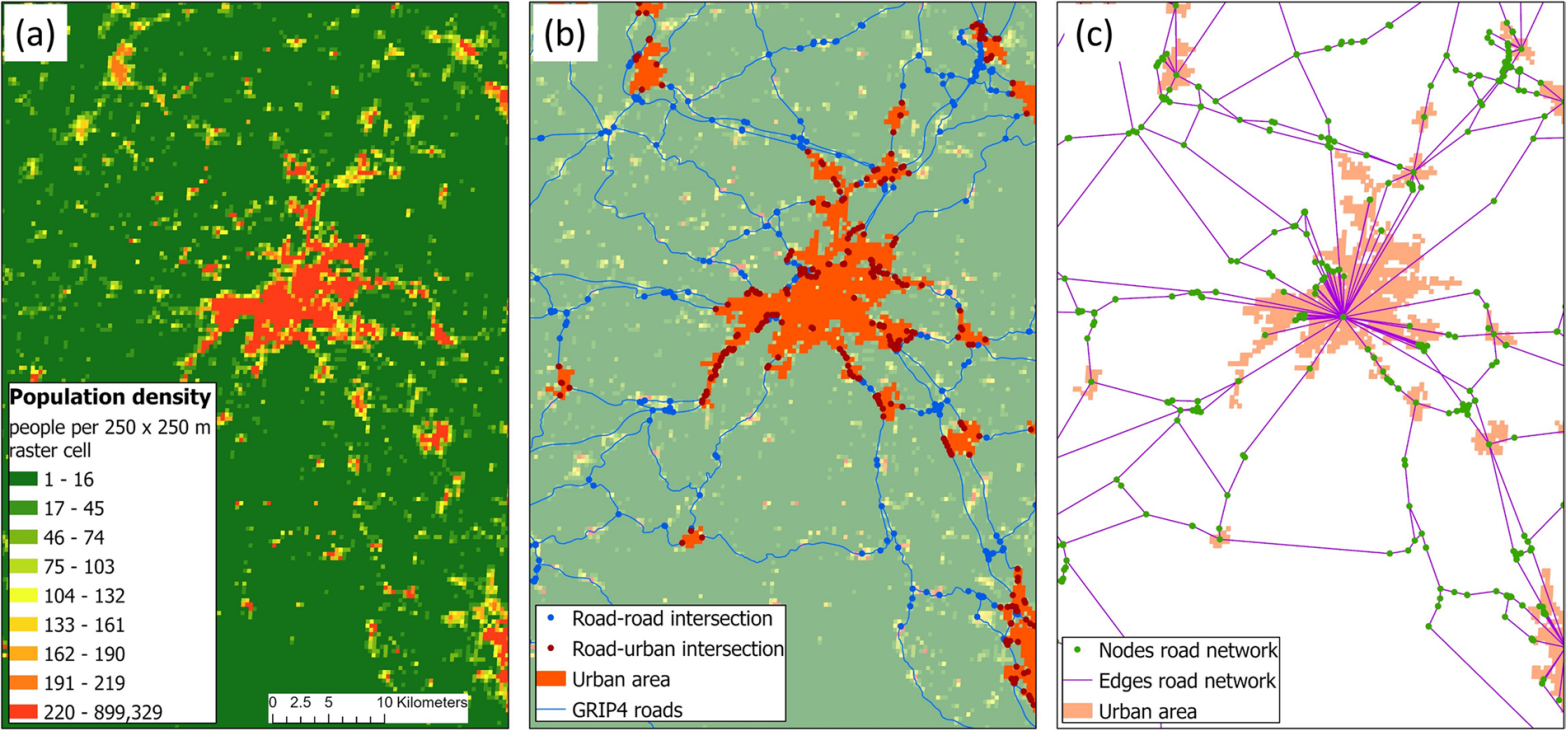
Overview of the analysis steps to create the road networks. The steps are demonstrated in a region around the city of Bern in Switzerland. (**a**) The global population density raster GHS-POP contains the population size in each 250 × 250 m raster cell^41^. (**b**) Urban areas were defined from this raster, by selecting clusters of connecting raster cells with a minimum population density of 93.75 people per cell and a total cluster size of at least 2000 people. From the global road dataset GRIP4^39^, intersections among roads (road-road intersections) or intersections of roads with urban areas (road-urban intersections) were defined. (**c**) These intersections formed nodes in the road network. The edges were defined by roads connecting the nodes. Attributes were calculated for all edges as explanatory variables of Annual Average Daily Traffic.

In this study, we produced the first freely available global time-series of traffic volume estimates on extra-urban roads by combining several existing global datasets on roads^39,40^, population density^41^ and socio-economic indicators^42^ with a large collection of empirical AADT training data from all continents except Antarctica. To do this, we developed a network-based model to predict AADT (Fig. 1). In the first step of our analysis, we delineated urban areas in the Global Human Settlement Layer GHS-POP^41^ (Fig. 1a) adapting the ‘degree of urbanisation’ classification scheme^43^ (Fig. 1b). All roads outside of these urban areas were considered extra-urban roads. Secondly, we constructed road networks for the years 1975, 1990, 2000 and 2015 in which the nodes were either urban areas or road intersections, while the edges represented all extra-urban highways, primary roads, and secondary roads in the recent GRIP4 roads dataset^39^ (Fig. 1c). We reconstructed the roads for 1975 and 1990 by combining the GRIP4 dataset^39^ with the older Vmap0 dataset^40^. For each road section, we calculated a set of explanatory variables for AADT, consisting of road characteristic, demographic, socio-economic and network topological variables. For 3% of the road sections, we also collected empirical AADT values from national traffic count datasets for the year 2015, which served as response variable in our model. We then trained a Quantile Regression Forest (QRF) model^44^, a variation to Random Forest models, for the year 2015 with 80% of the empirical AADT values (training set). With the trained model, we subsequently made predictions of AADT for every extra-urban road section for the four time-steps. Finally, we performed a hold-out validation of the 2015 predictions with the remaining 20% of the empirical AADT values (validation set) as well as an out-of-sample validation of the 1975 predictions with empirical AADT values from two countries that were not used in the calibration of the models. Both validations showed that the AADT estimates had a good accuracy.

The produced AADT time-series can be used for a variety of purposes. Spatially explicit data on traffic volumes can be used, for instance, to develop a system understanding of the effects of city and road network configurations on the connectivity in habitat networks^45^. Maps of AADT can also be used to pinpoint locations for wildlife under- or overpasses to ensure habitat connectivity across roads^46^. Roads with an AADT of 10 000 are regarded as an insurmountable barrier for animal movement^10^ and it thus makes sense to target roads with an AADT above this threshold to implement connectivity measures. Further potential uses of our AADT predictions are to assess the noise pollution by traffic across a region^47^ or to determine hotspots of pollutants in runoff from roads^32^. Traffic exposure maps, such as the ones produced by Madadi, *et al*.^48^ and Pratt, *et al*.^9^, are a useful tool to determine to what degree locations are affected by noise or pollutants from traffic. Our time-series of traffic volumes allows to make statements about the development of such effects e.g.^47^. The lack of large-scale road traffic data is an important reason why traffic volumes are not given adequate attention in ecological, environmental and climate studies^17^. We hope that our dataset will trigger an increased consideration of traffic in such studies.

## Methods

There exists a wide variety of traffic flow modelling approaches, ranging from micro-scale models that simulate the behaviour of individual road users to macroscale models that estimate traffic variables in an aggregated way^49^. Given our large spatial scale and data limitations, we developed a macro-scale model, which has been used often for the prediction of AADT^16,35^.

The analyses were carried out in Python^50^ and R^51^. Python-packages that we used were arcpy^52^, igraph^53^, numpy^54^, pandas^55^, and rasterio^56^. We used the following R-packages: quantregForest^44^, randomForest^57^ and ggplot2^58^.

### Time series of urban areas

To define extra-urban areas, we first delineated urban areas from the Global Human Settlement Layer GHS-POP^41^. The GHS-POP dataset is a global raster time-series depicting human population density estimates in 250 × 250 m raster cells for the years 1975, 1990, 2000 and 2015 (Fig. 1a). To select urban areas from this raster, we adapted the “degree of urbanisation” classification scheme, which was developed by several international organisations and adopted by the UN Statistical Commission^43^. This classification scheme has the advantage that it is not based on administrative boundaries, but on the identification of population clusters in a population density raster, such as GHS-POP^43^.

In the degree of urbanisation scheme, a two-step procedure is used to classify urban areas (Dijkstra *et al*. 2021). First, all raster cells above a population density threshold are selected. Second, the total population size for clusters of adjoining and selected cells is calculated. Clusters above a population size threshold are considered urban areas. The scheme thus depends on two thresholds: a cell-specific population density threshold and a cluster-specific population size threshold. As population density threshold, we adopted the threshold for cities from the degree of urbanisation scheme, which is 1 500 people per km^2^ (i.e. 93.75 people per 250 × 250 m raster cell). In this scheme, all raster cells with a population density below this threshold are considered semi-dense or rural area^43^, which we considered to be “extra-urban” area. As population size threshold, we took a threshold of 2 000 people following the definition of a town by Forman^59^, p. 4, who writes: “a town is a compact mainly residential area in agricultural or natural land that contains about 2 000 to 30 000 residents…”. Although the population size threshold for cities is 50 000 people in the degree of urbanisation scheme^43^, we chose for this lower threshold to also include high-density towns as urban area (Fig. 1b). We applied this classification to the GHS-POP raster layers for the four time-steps, to obtain a time-series of urban areas. We defined extra-urban areas as all terrestrial raster cells in GHS-POP that were not classified as urban area (i.e. city or high-density town). With this approach to define urban areas, we delineated 192 830, 252 150, 266 022, and 298 602 urban areas for the years 1975, 1990, 2000 and 2015, respectively. The delineated urban areas were nodes in the extra-urban road networks. The identified urban areas are also supplied as polygon shapefiles for each time step (see section “Data Records”).

### Time series of extra-urban road networks

Before we could construct the road networks for the different time-steps, we had to reconstruct historical roads. This was a challenge, as there are very few complete and freely available global road datasets^39^, let alone time series of such datasets. Due to this data shortage, the time series of extra-urban roads consisted of two time-steps (i.e. ≤1990 and ≥2000). For the years 2000 and 2015, we used the GRIP4 global road dataset^39^, which is one of the most complete and harmonised global road datasets publicly available. For the years 1975 and 1990, we approximated the historical road networks by combining the roads in the GRIP4 dataset^39^ with those in the Vmap0 dataset predating 1997^40^.

More specifically, to reconstruct the roads for 1975 and 1990, we downgraded (i.e. change a road to a lower category) the road type of GRIP4 roads that were either absent or had a lower category in the Vmap0 dataset^40^. Highways and primary roads in GRIP4 that were not classified as such in Vmap0, were downgraded to, respectively, primary road, or secondary road. Roads that were not present in Vmap0 were downgraded to secondary road. Secondary roads in GRIP4 were not further downgraded. As Vmap0 has a lower spatial accuracy than GRIP4^39^, we buffered all Vmap0 roads with 1500 m. If 90% of the length of a GRIP4 road section was overlapping this buffered area, the road section was linked to its Vmap0 counterpart. This strategy of reconstructing historical roads was chosen for several reasons. First, there are strong regional differences in the completeness of the road mapping in Vmap0 and comparable datasets^39^. Removing all roads from GRIP4 that were missing in Vmap0 would thus result in a severe underrepresentation of the historical roads in certain regions. Second, several studies have found that many major roads were once created by upgrading lower class roads e.g.^60,61^ and, hence, a downgrading going back in time makes sense. Third, as we found that the road type was an important explanatory variable for AADT (see section “Quantile regression forest (QRF) model”), we argue that changing the road type was the best compromise between leaving the roads unchanged over time and completely removing roads. Despite these justifications, this approach of reconstructing historical roads is not ideal, due to spatial variability in the level of detail of the Vmap0 dataset^39^ as well as the differing spatial accuracies of the two datasets. Nevertheless, given the plausibility of the historical traffic volume maps and the satisfying results of the out-of-sample validation (see section “Technical validation”), we believe that the historical road reconstruction is of sufficient quality to be useful in further studies.

To obtain the extra-urban roads from the above road time series, we extracted all the primary roads, secondary roads and highways that were not within an urban area (see section “Time series of urban areas”; Fig. 1b). For each extra-urban road section, we estimated travel speeds. We set the speed limits on highways, primary roads, and secondary roads to 120, 100 and 80 km*h^−1^, respectively. As the effective speeds on roads are usually lower than the speed limits, we applied a transformation developed by Gao, *et al*.^62^: effective speed = 0.942*speed limit − 9.0618. This led to effective speeds of 104, 85 and 66 km*h^−1^ on highways, primary roads, and secondary roads, respectively. Combined with the length of a road section, the travel time along each road section was calculated in minutes. The minimum travel time on each road section was set to 1 minute to facilitate subsequent calculations. We did not consider changes in travel times over the years.

To determine the nodes in the extra-urban road network, we calculated all intersections between roads (i.e. road-road intersections) as well as intersections of roads with a boundary of an urban area (road-urban intersections; Fig. 1b). A road-road intersection was created wherever two roads intersected or where two road sections met. Intersecting roads that in reality are not a crossing (e.g. bridges and underpasses) were not considered, but it is anyway unknown how accurate they are represented in the GRIP4 dataset. All the road-urban intersections for a certain urban area obtained the same node-identifier and coordinates (i.e. the centroid of the urban area; Fig. 1b). As intra-urban roads were not included in the road network, the size of the entire road network was strongly reduced enabling efficient computations. An edge was added to the road network between any two nodes that were connected by a highway, primary road, or secondary road. The calculated travel times on each road section was added as attribute to the edges.

Our final 2015 road network consisted of 2 539 301 nodes (road-road and road-urban intersections) and 3 300 765 edges. According to our definition of extra-urban roads, we found that the GRIP4 dataset contained 7 250 674 km of extra-urban highways, primary roads, and secondary roads in 2015, which corresponds to 89.5% of the total length of these road types in GRIP4.

For some analyses, such as habitat connectivity analyses, information on road tunnels is ideally required (i.e. roads in tunnels do not impair habitat connectivity). Yet, tunnels are not an attribute in the GRIP4 road database^39^. In some countries this could be a considerable bias, but for most countries it is expected that road tunnels are not particularly prevalent, e.g. in Europe, only 1.65% of major roads are in a tunnel^63^.

### Explanatory variables

For each edge in the road network, we calculated a range of explanatory variables for AADT. The list of potential variables was determined, on the one hand, by a literature analysis of AADT prediction studies, and, on the other hand, by the availability of global data for the relevant time steps. Making use of several literature reviews^16,35^, we identified four groups of explanatory variables for AADT: variables quantifying the human population, variables describing road characteristics, road network metrics, and variables quantifying socio-economic conditions. We tested 89 explanatory variables (the complete list is included in Supplementary Table 1), which we reduced with several steps (see section” Quantile Regression Forest (QRF) model”) to a final list of 13 variables (Table 1), which we will discuss here in more detail. All explanatory variables were calculated for each edge in the road network in each of the four time steps.

**Table 1:** Final selection of explanatory variables for Average Annual Daily Traffic. The variables are calculated for each edge in the road network.

Abbreviation	Category	Description	Data Source	Calculation
eMeanPop4ord	population density	Urban population density closely surrounding an edge.	GHS-POP (Florczyk *et al*. 2019)	Mean of the total population surrounding nodes *a* and *b*. Total population in a node is calculated by summing the population in the 4th order urban areas.
eMeanPop22ord	population density	Urban population density in a large area around an edge.	GHS-POP (Florczyk *et al*. 2019)	Mean of the total population surrounding nodes *a* and *b*. Total population in a node is calculated by summing the population in the 22nd order urban areas.
GP_RTP	road characteristics	Road type: highway, primary road or secondary road.	GRIP4 (Meijer *et al*. 2018), Vmap0 (NIMA 1997)	GP_RTP attribute from GRIP4. For 1975 and 1990, adapted with Vmap0
eMeanCirclePop	road characteristics	Proxy for land use surrounding a road.	GHS-POP (Florczyk *et al*. 2019)	The mean of the population in a 2 km radius around nodes *a* and *b*
eDiffCirclePop	road characteristics	Proxy for a land use change gradient along a road.	GHS-POP (Florczyk *et al*. 2019)	Absolute difference between the populations in a 2 km radius around nodes *a* and *b*
eBetw240	road network metrics	Number of long distance trips passing through an edge	GRIP4 (Meijer *et al*. 2018), Vmap0 (NIMA 1997)	Edge betweenness centrality (weight = travel time) only considering nodes at 240 min travel time
eBetw60	road network metrics	Number of short distance trips passing through an edge	GRIP4 (Meijer *et al*. 2018), Vmap0 (NIMA 1997)	Edge betweenness centrality (weight = travel time) only considering nodes at 60 min travel time
eDiffBetw60	road network metrics	Relative difference in short distance trips passing through the nodes on either side of an edge. Potentially informative for dead-end roads	GRIP4 (Meijer *et al*. 2018), Vmap0 (NIMA 1997)	Absolute difference between the betweenness centrality (weight = travel time) of nodes *a* and *b* relative to the mean betweenness in both nodes. The betweenness of a node is calculated only by considering nodes at 60 min travel time
eMeanStrength	road network metrics	Proxy for the isolatedness of nodes on either side of an edge	GRIP4 (Meijer *et al*. 2018), Vmap0 (NIMA 1997)	The mean of the average travel time of nodes *a* and *b* to their 1st order neighbors
eMeanBetw60	road network metrics	The mean number of short distance trips passing through the nodes on either side of an edge	GRIP4 (Meijer *et al*. 2018), Vmap0 (NIMA 1997)	Mean betweenness centrality (weight = travel time) of nodes *a* and *b*. The betweenness of a node is calculated only by considering nodes at 60 min travel time
eMeanNei4ord	road network metrics	The density of nodes surrounding an edge	GHS-POP (Florczyk *et al*. 2019)	Mean of the number of 4th and lower order nodes of nodes *a* and *b*
eMeanGDP	socio-economic	Mean gross domestic product of an edge	GDP raster (Kummu *et al*. 2018)	Mean gross domestic product of nodes *a* and *b*
eMeanHDI	socio-economic	Mean human development index of an edge	HDI raster (Kummu et al. 2018)	Mean human development index of nodes *a* and *b*

An overview of all tested explanatory variables can be found in Supplementary Table 1. Nodes *a* and *b* refers to the nodes on either side of an edge.

Since the presence of people is a prerequisite of traffic, it is not surprising that variables quantifying the human population in the surrounding of a road were found to be important to explain AADT^35,64^. Therefore, we included one population density variable that quantified the population in urban areas closely around a road section (eMeanPop4Ord) and one that quantified it at a larger distance around a road section (eMeanPop22Ord, Table 1).

As road characteristic, we included the road type, which was taken directly from the GRIP4 dataset^39^ for the years 2000 and 2015, or from the potentially downgraded roads for the years 1975 and 1990 (see section “Time series of extra-urban road networks”). Another variable that has regularly been found to influence traffic is the land use surrounding a road^35^, where land use usually refers to different types of urban land use^16^. As the land use is strongly influenced by the population density, we calculated the total population in a 2 km radius around each node using the GHS-POP dataset^41^. For each edge in the road network, we summarised this information in two variables: eMeanCirclePop and eDiffCirclePop (Table 1). Whereas the population density variables eMeanPop4Ord and eMeanPop22Ord summarise the population in urban areas at different proximities around a road, these variables only assess the population directly around a road section in both urban and extra-urban settings.

Various network-based studies have found that network metrics (e.g. betweenness centrality, page rank, degree) can also be useful explanatory variables of traffic volumes^37,65^. In our final selection of explanatory variables, we included variables based on the edge- (eBetw60, eBetw240) and node-betweenness (eMeanBetw60, eDiffBetw60; Table 1), which indicate the number of shortest routes that pass through an edge or through the nodes on either side of the edge, respectively. Travel times were used as edge weight to calculate these shortest paths. As a measure of isolatedness of a node, we also calculated the mean travel time from the respective node to its direct neighbours. To calculate the edge attribute, this value was averaged for the nodes on either side of an edge (eMeanStrength; Table 1).

As correlations have been found between socio-economic conditions and traffic volumes^64^ or car ownership^66^, we calculated the mean Gross Domestic Product (GDP) per capita and mean Human Development Index (HDI) for each edge in the road network (eMeanGPD, eMeanHDI). This data was extracted from Kummu, *et al*.^42^, who created global raster layers of GDP per capita (purchasing power parity) and HDI for the year 1990, 2000 and 2015. To estimate the values for 1975, we extrapolated the trend in the latter years with log-linear regression for GDP^67^ and logistic regression for HDI^68^.

### Response variable: empirical AADT values

**Fig. 2:**
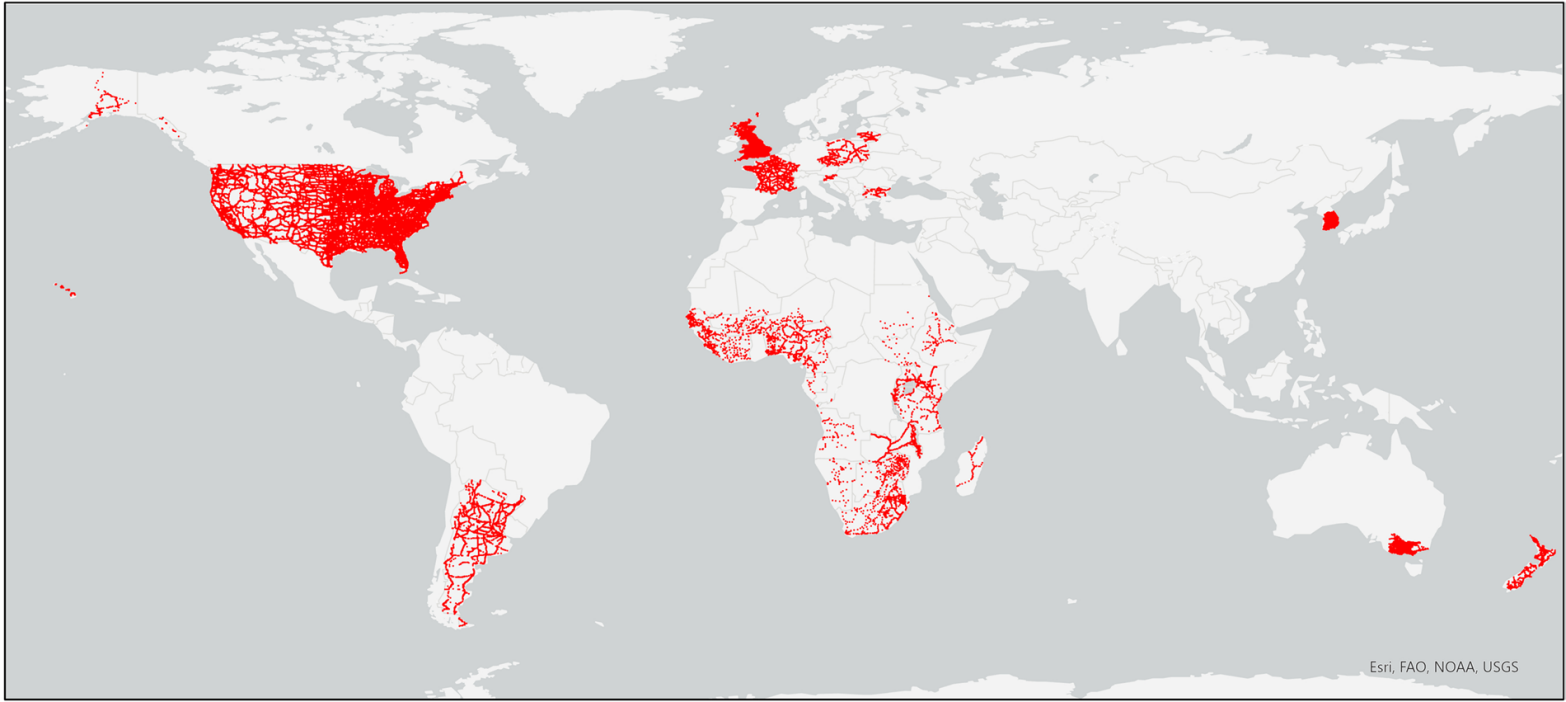
Map of the empirical Annual Average Daily Traffic (AADT) values (red dots) that could be assigned to edges in the road network.

As response variable, we collected spatially explicit datasets (i.e. line or point shapefiles) with empirical AADT values from around the year 2015. We found such datasets for 46 countries (Table 2) located in all continents (except Antarctica; Fig. 2). We made a considerable effort to find datasets from the year 2015 but, for some countries, had to resort to datasets from earlier or later years (Table 2). We assumed that these earlier or later datasets were still representative of localised traffic in 2015.

**Table 2:** Sources of empirical AADT values used to train the Quantile Regression Forest model.

Continent	Country	Year published	Organisation	URL	Nr. AADT values	Nr. highways	Nr. primary roads	Nr. secondary roads
Africa	Angola, Benin, Botswana, Burkina Faso, Burundi, Cameroon, Democratic Republic of the Congo, Eritrea, Ethiopia, Gabon, Guinea, Kenya, Lesotho, Liberia, Madagascar, Malawi, Mali, Mauritania, Namibia, Niger, Nigeria, Rwanda, Senegal, Sierra Leone, South Africa, South Sudan, Sudan, Swaziland, Tanzania, The Gambia, Togo, Uganda, Zambia, Zimbabwe.	2009	Africa Infrastructure Country Diagnostics (AICD), World Bank Group 2009	https://datacatalog.worldbank.org/	1794	263	925	606
Asia	South Korea	2012	Korea Institute of Construction Technology, Ministry of Land, Infrastructure and Transport, Traffic Monitoring System	Shapefile: http://www.biz-gis.com/index.php?mid=pds&document_srl=147626&category=5160. Raw data: http://www.road.re.kr/itms/itms_1.asp	2790	352	1337	1101
Europe	Bulgaria, Czech Republic, Lithuania, Poland, Slovenia	2015	United Nations Economic Commission for Europe	https://unece.org/traffic-census-2015-0	778	428	314	36
	France	2015	Ministry of Ecological Transition (French: Ministère de la Transition écologique)	https://www.data.gouv.fr/fr/datasets/r/65f579c8-e8de-4d56-80dc-2f7573f6291a	1570	1214	321	35
	Great Britain	2015	Department for Transport	https://roadtraffic.dft.gov.uk/downloads	9937	4837	4298	802
North America	United States of America	2012	Department of Transportation’s Federal Highway Administration	https://www.fhwa.dot.gov/policyinformation/hpms/shapefiles_2017.cfm	75120	17269	31495	26356
Oceania	Australia (Victoria)	2020	Department of Transport, Victoria State Government	https://vicroadsopendata-vicroadsmaps.opendata.arcgis.com/datasets/vicroadsmaps::traffic-volume/about	3018	390	1449	1179
	New Zealand	2015	State Highway Management Division	https://hub.arcgis.com/datasets/NZTA::state-highway-traffic-monitoring-sites/about	1316	277	1029	10
South America	Argentina	2017	Directorate of the National Transport Observatory (Spanish: Dirección de Observatorio Nacional de Transporte)	https://datos.gob.ar/dataset/transporte-tmda	3005	36	2737	232
				**Total**	**99328**	**25066**	**43905**	**30357**

The last four columns refer to the number of edges (i.e. road sections) in the road network that had an empirical AADT value assigned to them.

Empirical AADT values were assigned to the closest GRIP4 road section that was within a certain maximum distance from the sample point. These maximum distances were set to 100, 200 or 250 m depending on the spatial accuracy of the AADT shapefile. We manually adjusted some of the AADT shapefiles to improve overlap with the GRIP4 dataset. If more than one AADT value could be matched to a road section, the mean of the values was taken. Although most datasets contained AADT values derived from vehicle count data, some datasets also contained some modelled estimates of AADT. Most of the AADT datasets were publicly available, and for some we were granted permission for their use by the owners.

The AADT values from three countries had a relatively high number of 0 values (France: 396; New Zealand: 51; USA: 37). Upon visual inspection of the locations with AADT = 0, we noticed that the vast majority of these locations had neighbouring AADT values that were considerably higher. Therefore, we considered these locations as misspecified or missing data points and only considered road sections with AADT values larger than 0 in subsequent analyses.

Our 2015 road network finally contained 99 328 edges with empirical AADT values assigned to them, which corresponds to 3.0% of the edges in the entire road network. Each of the considered road classes was well represented in this training data (Table 2).

### Quantile regression forest (QRF) model

To relate the explanatory variables to AADT, we used a QRF model^44^, which is a variation to the well-known Random Forest model. Various studies have found that Random Forest models outperform other methods when predicting AADT^16,35^. However, whereas Random Forest and many other regression techniques only provide a mean predicted value as output, QRF is capable of calculating prediction intervals by modelling the complete conditional distribution of the response variable^44^. These intervals express the upper and lower limits between which a true value is likely to fall and, thus, gives an indication of the reliability of a single prediction^44^. Due to the right-skewed distribution of the empirical AADT values and following other AADT prediction studies e.g.^38,69,70^, we transformed AADT with the natural logarithm before fitting the QRF models.

Before training our final QRF model, we tuned the following hyperparameters: number of trees (*ntree*), number of randomly selected variables at each split (*mtry*), minimum size of terminal nodes (*nodesize*), and size of samples to draw (*sampsize*)^57^. Making use of the R-package TuneRanger^71^, we found that *mtry* = 5, *nodesize* = 4 and *sampsize* = 0.871 (i.e. 87.1% of the training data) were optimal QRF hyperparameter settings for our dataset. We found that training and validation accuracy converged well up to 2500 trees, stayed practically constant till 3500 trees and then started to diverge (i.e. overfitting) with larger numbers of trees. Therefore, we set *ntree* = 2500. For the other arguments in the quantregForest^44^ and randomForest^57^ R-functions, we used default settings.

**Fig. 3:**
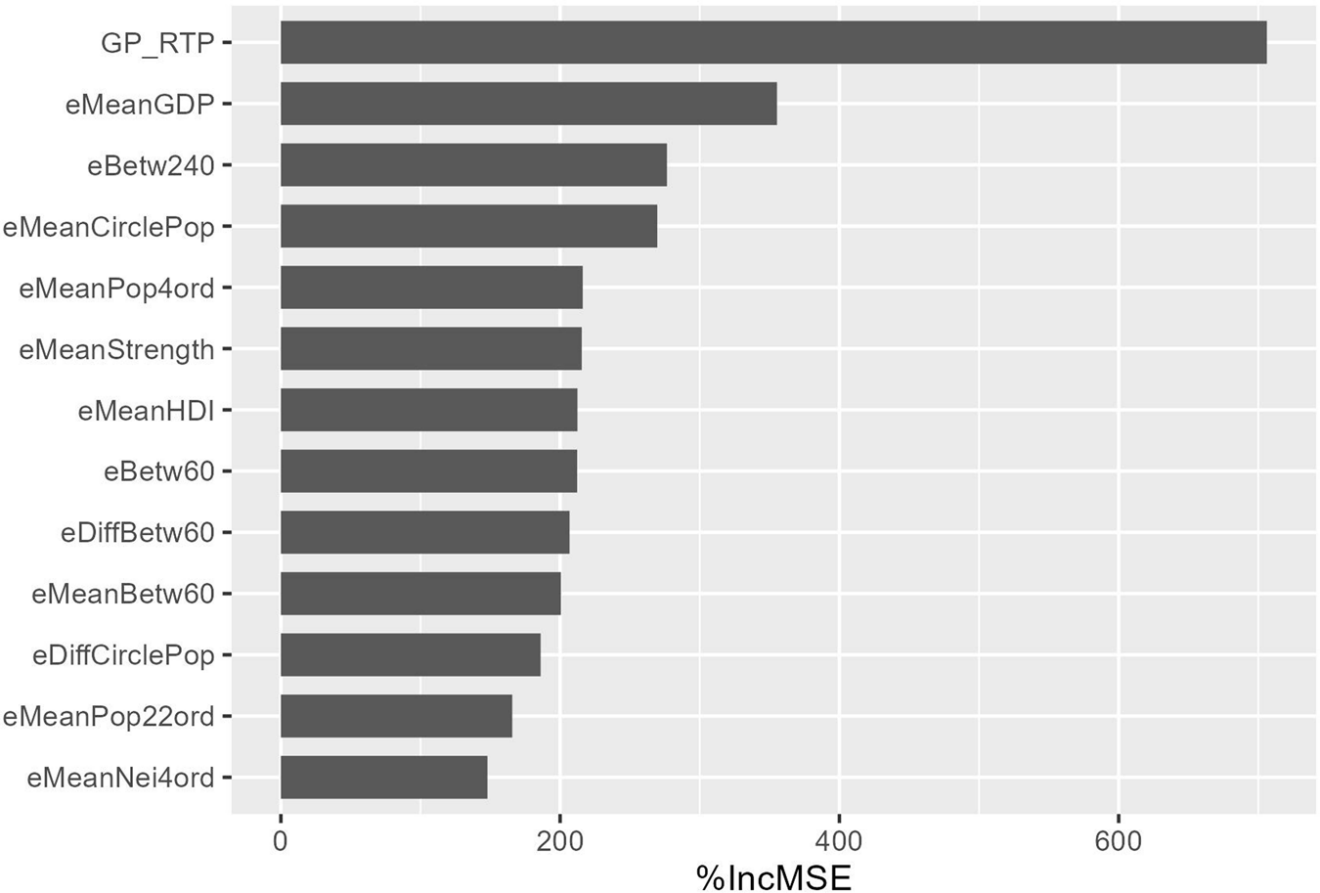
Importance of the explanatory variables in the Quantile Regression Forest model. The value on the x-axis, “%IncMSE”, is an indicator for variable importance.

To reduce the initial set of explanatory variables (Supplementary Table 1) to our final selection (Table 1), we selected variables based on their importance in the model, the linear correlation among the variables and the percentage of explained variance of the QRF model. None of the finally selected explanatory variables were strongly correlated (Pearson *r ≤ *0.66). Also, the explained variance of the QRF model fitted with the selected variables was comparable to that of the full model. The importance of the 13 explanatory variables in the final model, indicated that road type (GP_RTP) was clearly the most important explanatory variable in the QRF model, followed by the variables eMeanGDP, eBetw240 and eMeanCirclePop (Fig. 3 & Table 1).

**Fig. 4:**
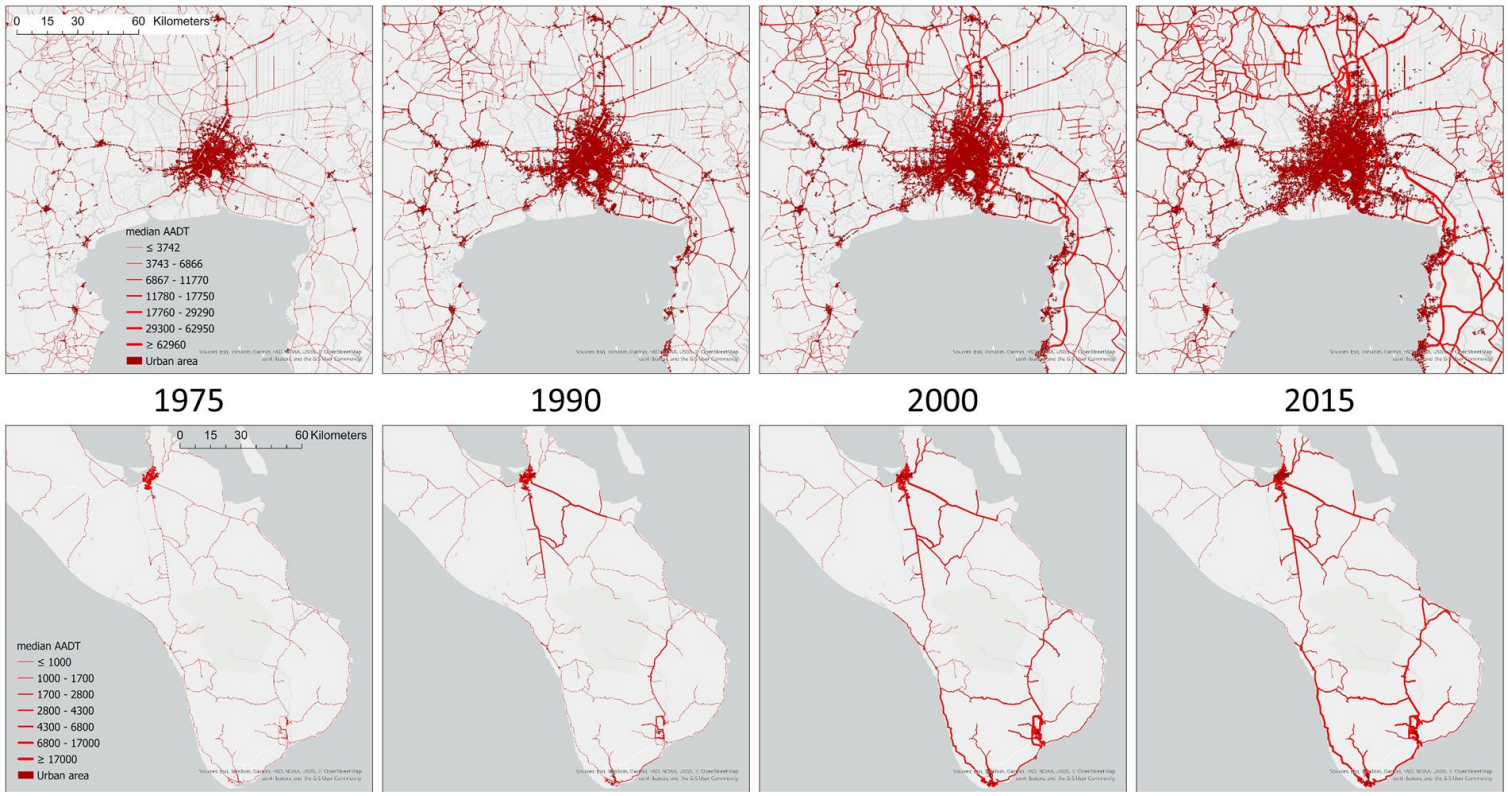
Maps to illustrate the predictions of median Annual Average Daily Traffic (AADT) on extra-urban roads for the years 1975, 1990, 2000 and 2015. The top map series is for the region around the city of Bangkok in Thailand, whereas the bottom series depicts the southern tip of the Baja California Peninsula in Mexico (the main city in the maps is La Paz in the North). The expansion of the urban area as well as the growth in traffic volumes can be clearly seen in both map series.

We used the trained QRF model to predict the median AADT value as well as the 5% and 95% prediction intervals for each edge in the road network. In other words, the model predicted that there was a 90% chance that the true AADT value of an edge would be between these intervals. A visual comparison of the time series for two arbitrarily chosen regions in the world clearly showed the increase in traffic volume in these two regions in the period 1975 to 2015 (Fig. 4). The growth of the urban area during this period can also be observed. The general patterns of traffic flows also seem realistic, with through roads that connect urban areas receiving more traffic than side roads.

### Mean AADT and growth per country

To summarise our findings, we calculated the mean AADT per country for each of the time steps. The country boundaries were taken from the geoBoundaries dataset^72^. The mean AADT was calculating by weighting the median AADT predictions of each road section by its length. This way relatively short road sections with a relatively high or low AADT would not have a disproportionate effect on the final mean. From these mean AADT values and for each time period, we also calculated the compound annual growth, which is the annual percentage of growth that would be necessary to get from the AADT in one time step to the next. We calculated compound growth for the time periods 1975–1990, 1990–2000, and 2000–2015 as well as for the entire time period 1975–2015. A table with these statistics per country is made available (see section “Data Records”)^73^.

## Data Records

The data produced in this study are available in a zip-file via the ETH research collection: 10.3929/ethz-b-000666313^73^

The zip-file contains the table Country_meanAADT&Growth_20251104.csv, which lists for each country the weighted mean AADT on extra-urban roads for the years 1975, 1990, 2000 and 2015 as well as the compound growth in mean AADT for the time periods 1975–1990, 1990–2000, 2000–2015 and 1975–2015.

The zip-file furthermore contains folders for each of the years 1975, 1990, 2000 and 2015. Each folder contains a vector map of extra-urban roads (line shapefile format; GRIP4_ExSet_XXXX_AADTpred_YYYYMMDD.shp where XXXX refers to the year and YYYYMMDD to the production date) with the predicted median AADT as well as 5% and 95% prediction intervals for each road section, vector maps of urban areas (polygon shapefile format; GHS_POP_Clump_XXXX.shp) delineated according to the definition used in this study, and the Python and R code used to create the road networks and perform the analyses. A README.txt file provides further details of the contents of the zip-file.

## Technical Validation

We performed two types of model validation: (1) a hold-out validation of the AADT predictions of 2015 and (2) an out-of-sample validation of the AADT predictions of 1975. To get an impression of the representativeness of our training data, we also assessed how well the explanatory variables in the training dataset overlapped with the ranges of values found in the complete road networks from all years.

### Hold-out validation

For the hold-out validation, we randomly assigned 20% of our empirical AADT values to a validation set (i.e. 19 863 observations). The remaining 80% of the response data was used to train the QRF model. To determine the quality of the predictions for the validation set, we used mean square error (MSE), the percentage of explained variance (pseudo-R^2^) and the percentage of observations that fall within the prediction intervals.

**Fig. 5:**
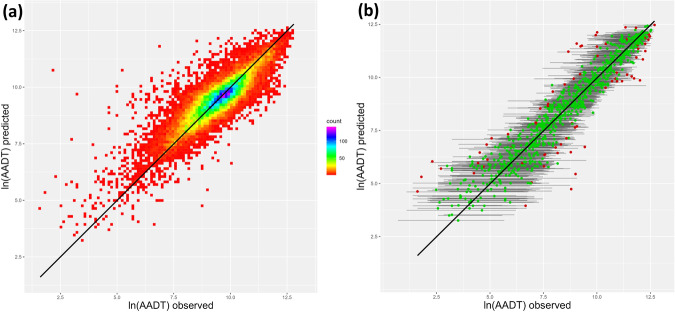
Plots of the observed and predicted median ln(AADT) values of the validation set. The black lines indicate perfect prediction (i.e. observed = predicted). (**a**) A density plot of all observations showing that the majority of observations is close to the line of perfect prediction. (**b**) Scatterplot for a selection of AADT values from the validation set to illustrate the prediction intervals. The grey lines indicate the 90% prediction intervals. The colours of the dots indicate whether an observed AADT is within (green) or outside (red) the prediction interval. AADT = Annual Average Daily Traffic.

After training the model, the training and validation pseudo-R^2^ were very similar: 0.7418 and 0.7407, respectively. This indicates that 74.07% of the variance in the validation set could be explained by the model. The model does not seem to be overfitting considering the very small difference between the training and validation pseudo-R^2^ values. A visual comparison of the observed and predicted ln(AADT) values also confirms the good fit of the model (Fig. 5), with most observations being evenly distributed around and close to the line of perfect prediction (i.e. predicted = observed; black line in Fig. 5a). The prediction intervals seem to be correctly estimated by the QRF model, as we found that 92.1% (18 301 out of 19 863 observations in the validation set) of the observed AADT values fell within the 90% prediction intervals (exemplified in Fig. 5b).

The model fit (pseudo-R^2^) was relatively high compared to those found in other AADT prediction studies^34,35^. Although most of the studies covered in the reviews of Das and Tsapakis^35^ and Baffoe-Twum, *et al*.^34^ were carried out at regional or national scale, the reported R^2^ values, with some exceptions, were lower than or similar to that of our model.

### Out-of-sample validation

For the out-of-sample validation, we obtained two AADT datasets derived from empirical traffic counts for 1975 from two countries of which no data was used to train the QRF: Switzerland and the Netherlands. For Switzerland, we obtained AADT measurements for 1975 in a table format (Swiss Federal Roads Office; https://www.astra.admin.ch/astra/en/home/documentation/data-and-information-products/traffic-data/data-and-publication/swiss-automatic-road-traffic-counts--sartc-.html), which we linked to the coordinates of counting stations and then transferred to our road network. For the Netherlands, we obtained digitised maps of AADT (work day averages) for road sections in 1975 (Centraal Bureau voor de Statistiek; https://open.rws.nl/open-overheid/onderzoeksrapporten/@165173/algemene-verkeerstellingen). We georeferenced these maps and then transferred a random selection of these AADT values to our road network. For Switzerland and the Netherlands, we finally obtained, respectively, 28 and 87 empirical AADT values that could be linked to road sections in our 1975 road network. We compared these true AADT values with the predicted ones by calculating the linear correlation and the percentage of observations that fell within the prediction intervals.

**Fig. 6:**
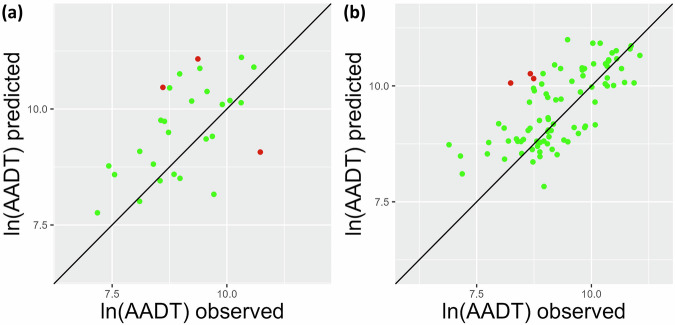
Scatterplots of the 1975 observed and predicted ln(AADT) in (**a**) Switzerland and (**b**) the Netherlands. The black lines indicate perfect prediction (i.e. observed = predicted). The dot colours indicate whether an observed AADT is within (green) or outside (red) the 90% prediction interval. AADT = Annual Average Daily Traffic.

The out-of-sample validation showed that the model was able to predict historical AADT values with a high level of accuracy (Fig. 6). For both Switzerland and the Netherlands, the Pearson *r* correlation between predicted and observed ln(AADT) values was highly significant (*p* < 0.005) and positive (0.55 and 0.70, respectively). The percentage of observations that fell within the 90% prediction interval was 89.3% and 96.5% for, respectively, Switzerland (Fig. 6a) and the Netherlands (Fig. 6b). These percentage dropped to 63.6% and 77.4% when using the 2015 predictions, indicating that the 2015 predictions are not a good proxy for AADT in 1975.

### Representativeness of training data

We found that the range of values covered by the explanatory variables in the training dataset gave a good representation of the values found in the complete road networks from all years (Table 3). For most explanatory variables and years, all or nearly all (≥97%), of the road sections had values that fell within the range of the training set (Table 3). This indicates that the training data was a representative sample of global roads and their AADT. Only for GDP and HDI, the range of values in the training dataset did not cover the entire range found in all road sections in the 1975 road network (78 and 79% covered, respectively; Table 3). This may have been due to the fact the GDP and HDI have increased since 1975 but could also be a result of the extrapolation that we carried out to estimate GDP and HDI values for 1975. Although we used published extrapolation methods for these indicators^67,68^, the estimated values could still have been biased, especially because the extrapolation was only based on three data points (i.e. 1990, 2000 and 2015).

**Table 3:** The minimum (Train min.) and maximum (Train max.) values of the different explanatory variables in the training set and the proportion of road sections that fall within this range for each of the years.

Explanatory variable	Train min.	Train max.	2015	2000	1990	1975
eMeanPop4ord	0.0	22801592.7	0.99	1.00	1.00	1.00
eMeanPop22ord	0.0	27749218.4	0.97	0.98	0.99	1.00
GP_RTP (categorical)	1	3	1.00	1.00	1.00	1.00
eMeanCirclePop	0.0	267373.8	1.00	1.00	1.00	1.00
eDiffCirclePop	0.0	319342.3	1.00	1.00	1.00	1.00
eBetw240	0.0	227269171.1	1.00	1.00	1.00	1.00
eBetw60	0.0	13576521.0	1.00	1.00	1.00	1.00
eDiffBetw60	0.0	2.0	1.00	1.00	1.00	1.00
eMeanStrength	1.0	240.2	1.00	1.00	1.00	1.00
eMeanBetw60	0.0	22213730.8	1.00	1.00	1.00	1.00
eMeanNei4ord	2.0	4016.0	0.99	1.00	1.00	1.00
eMeanGDP	692.7	92755.0	0.99	0.98	0.99	0.78
eMeanHDI	0.4	1.0	1.00	0.99	0.98	0.79

A detailed explanation of these explanatory variables can be found in Table 1. An overview of all tested explanatory variables can be found in Supplementary Table 1.

## Usage Notes

Whereas the GRIP4 global road dataset is one of the most complete and harmonised global road datasets publicly available, the Vmap0 road dataset shows considerable variability in the accuracy and completeness of the road mapping between regions^39^. The roads for the years 2000 and 2015 were taken directly from GRIP4, but we used the Vmap0 dataset in the reconstruction of the roads for the years 1975 and 1990. Therefore, before using the AADT predictions in further analyses, users of our dataset should assess the reliability of the reconstructed historical road networks for their area of interest, particularly for the years 1975 and 1990.

## Supplementary information

Supplementary Table 1

## Data Availability

The Python and R code used to create the road networks and perform the analyses is provided together with the output data (see section “Data Records”).
