# Correction: A global time series of traffic volumes on extra-urban roads

**DOI:** 10.1038/s41597-026-06764-9

**Published:** 2026-02-09

**Authors:** Maarten J. van Strien, Adrienne Grêt-Regamey

**Affiliations:** Planning of Landscape and Urban Systems PLUS, ETH Zurich, Stefano-Franscini-Platz 5, CH-8093 Zurich, Switzerland

Correction to: *Scientific Data* 10.1038/s41597-024-03287-z, published online 8 May 2024.

Since the version of the article initially published, an error has been identified in the code used to generate traffic volume predications for 1975 and 1990. The predictions for 2000 and 2015 remain unaffected. The code has been corrected, and the following amendments have been made as a result.

In the “Time series of extra-urban road networks” section, the text “Highways and primary roads in GRIP4 that were either not present or not classified as such in Vmap0, were downgraded to, respectively, primary road, or secondary road. Secondary roads in GRIP4 were not further downgraded” has been amended to “Highways and primary roads in GRIP4 that were not classified as such in Vmap0, were downgraded to, respectively, primary road, or secondary road. Roads that were not present in Vmap0 were downgraded to secondary road. Secondary roads in GRIP4 were not further downgraded.” In the “Data Records” section, the file name “Country_meanAADT&Growth_20240325.csv” has been amended to “Country_meanAADT&Growth_20251104.csv” and the text “(line shapefile format; GRIP4_ExSet_XXXX_AADTpred_20240312.shp where XXXX refers to the year)” has been amended to “(line shapefile format; GRIP4_ExSet_XXXX_AADTpred_YYYYMMDD.shp where XXXX refers to the year and YYYYMMDD to the production date)”. In the “Out-of-sample validation” section, the text “we obtained digitised maps of AADT” has been amended to “we obtained digitised maps of AADT (work day averages)”; the text “27 and 87 empirical AADT values” has been amended to “28 and 87 empirical AADT values”; the text “highly significant (*p* < 0.001) and positive (0.60 and 0.70, respectively)” has been amended to “highly significant (*p* < 0.005) and positive (0.55 and 0.70, respectively)”; the text “The percentage of observations that fell within the 90% prediction interval was 85.2%” has been amended to “The percentage of observations that fell within the 90% prediction interval was 89.3%”; and the text “These percentage dropped to 63.3% and 77.4%” has been amended to “These percentage dropped to 63.6% and 77.4%”. Figs. 4 and 6 have also been updated and the original, uncorrected figures are available for comparison in the Supplementary information accompanying this amendment. These corrections have been made to the HTML and PDF versions of the article.

The corrected data has been uploaded to the ETH research collection and is accessible via the original URL (10.3929/ethz-b-000666313).

## Supplementary information

Original, uncorrected Figs. 4 and 6

