# Supplementary material for: A global time series of traffic volumes on extra-urban roads
Source: Sci Data. 2024 May 8;11:470. doi: 10.1038/s41597-024-03287-z (PMC11078983; doi:10.1038/s41597-024-03287-z)
Supplement: Supplementary file 1 — Supplementary Table 1 [file 41597_2024_3287_MOESM1_ESM.pdf]

Supplementary Table 1. Complete list of edge attributes in the road network that were tested as explanatory variables for Annual Average Daily Traffic. The terms "source" and "target" node refer to the nodes on either side of an edge and can be used interchangeably.

Abbreviation	Description
GP_RTP	Road type
time_min	Travel time (minutes)
eSPop1ord	Total population in all 1st order nodes of source node
eTPop1ord	Total population in all 1st order nodes of target node
eSNei1ord	Number of 1st order neighbors of source node
eTNei1ord	Number of 1st order neighbors of target node
eMeanPop1ord	Mean of eSPop1ord and eTPop1ord
edgeDiffPop1ord	Absolute difference between eSPop1ord and eTPop1ord
eMeanNei1ord	Mean of eSNei1ord and eTNei1ord
edgeDiffNei1ord	Absolute difference between eSNei1ord and eTNei1ord
eSPop4ord	Total population in all 4th order nodes of source node
eTPop4ord	Total population in all 4th order nodes of target node
eSNei4ord	Number of 4th order neighbors of source node
eTNei4ord	Number of 4th order neighbors of target node
eMeanPop4ord	Mean of eSPop4ord and eTPop4ord
edgeDiffPop4ord	Absolute difference between eSPop4ord and eTPop4ord
eMeanNei4ord	Mean of eSNei4ord and eTNei4ord
edgeDiffNei4ord	Absolute difference between eSNei4ord and eTNei4ord
eSPop7ord	Total population in all 7th order nodes of source node
eTPop7ord	Total population in all 7th order nodes of target node
eSNei7ord	Number of 7th order neighbors of source node
eTNei7ord	Number of 7th order neighbors of target node
eMeanPop7ord	Mean of eSPop7ord and eTPop7ord
edgeDiffPop7ord	Absolute difference between eSPop7ord and eTPop7ord
eMeanNei7ord	Mean of eSNei7ord and eTNei7ord
edgeDiffNei7ord	Absolute difference between eSNei7ord and eTNei7ord
eSPop10ord	Total population in all 10th order nodes of source node
eTPop10ord	Total population in all 10th order nodes of target node
eSNei10ord	Number of 10th order neighbors of source node
eTNei10ord	Number of 10th order neighbors of target node
eMeanPop10ord	Mean of eSPop10ord and eTPop10ord
edgeDiffPop10ord	Absolute difference between eSPop10ord and eTPop10ord
eMeanNei10ord	Mean of eSNei10ord and eTNei10ord
edgeDiffNei10ord	Absolute difference between eSNei10ord and eTNei10ord
eSPop13ord	Total population in all 13th order nodes of source node
eTPop13ord	Total population in all 13th order nodes of target node
eSNei13ord	Number of 13th order neighbors of source node
eTNei13ord	Number of 13th order neighbors of target node
eMeanPop13ord	Mean of eSPop13ord and eTPop13ord
edgeDiffPop13ord	Absolute difference between eSPop13ord and eTPop13ord
eMeanNei13ord	Mean of eSNei13ord and eTNei13ord
edgeDiffNei13ord	Absolute difference between eSNei13ord and eTNei13ord
eSPop16ord	Total population in all 16th order nodes of source node
eTPop16ord	Total population in all 16th order nodes of target node
eSNei16ord	Number of 16th order neighbors of source node
eTNei16ord	Number of 16th order neighbors of target node
eMeanPop16ord	Mean of eSPop16ord and eTPop16ord
edgeDiffPop16ord	Absolute difference between eSPop16ord and eTPop16ord
eMeanNei16ord	Mean of eSNei16ord and eTNei16ord
edgeDiffNei16ord	Absolute difference between eSNei16ord and eTNei16ord
eSPop19ord	Total population in all 19th order nodes of source node
eTPop19ord	Total population in all 19th order nodes of target node
eSNei19ord	Number of 19th order neighbors of source node
eTNei19ord	Number of 19th order neighbors of target node
eMeanPop19ord	Mean of eSPop19ord and eTPop19ord
edgeDiffPop19ord	Absolute difference between eSPop19ord and eTPop19ord
eMeanNei19ord	Mean of eSNei19ord and eTNei19ord
edgeDiffNei19ord	Absolute difference between eSNei19ord and eTNei19ord
eSPop22ord	Total population in all 22nd order nodes of source node
eTPop22ord	Total population in all 22nd order nodes of target node

Abbreviation	Description
eSNei22ord	Number of 22nd order neighbors of source node
eTNei22ord	Number of 22nd order neighbors of target node
eMeanPop22ord	Mean of eSPop22ord and eTPop22ord
edgeDiffPop22ord	Absolute difference between eSPop22ord and eTPop22ord
eMeanNei22ord	Mean of eSNei22ord and eTNei22ord
edgeDiffNei22ord	Absolute difference between eSNei22ord and eTNei22ord
eTPagerank	Pagerank (weight = time_min) of target node
eSPagerank	Pagerank (weight = time_min) of source node
eMeanPagerank	Mean of eTPagerank and eSPagerank
eDiffPagerank	Absolute difference between eTPagerank and eSPagerank relative to eMeanPagerank
eTMeanStrength	Mean travel time (minutes) from target node to neighboring nodes
eSMeanStrength	Mean travel time (minutes) from source node to neighboring nodes
eMeanStrength	Mean of eTMeanStrength and eSMeanStrength
eTBetw60	Betweenness centrality (weight = time_min) of target node only considering nodes at 60 min travel time
eSBetw60	Betweenness centrality (weight = time_min) of source node only considering nodes at 60 min travel time
eMeanBetw60	Mean of eTBetw60 and eSBetw60
eDiffBetw60	Absolute difference between eTBetw60 and eSBetw60 relative to eMeanBetw60
eMeanGDP	Mean gross domestic product of source and target node
eMeanHDI	Mean human development index of source and target node
eSCirclePop	Total population in a 2 km radius around source node
eTCirclePop	Total population in a 2 km radius around target node
eMeanCirclePop	Mean of eSCirclePop and eTCirclePop
eDiffCirclePop	Absolute difference between eSCirclePop and eTCirclePop
eBetw60	Edge betweenness centrality (weight = time_min) only considering nodes at 60 min travel time
eBetw240	Edge betweenness centrality (weight = time_min) only considering nodes at 240 min travel time
EucDist	Euclidean distance between nodes (m)
Length	Length of the road section (m)
DistRatio	Length/EucDist
