# Supplementary material for: Correction: A global time series of traffic volumes on extra-urban roads
Source: Sci Data. 2026 Feb 9;13:204. doi: 10.1038/s41597-026-06764-9 (PMC12886839; doi:10.1038/s41597-026-06764-9)
Supplement: Supplementary file 1 — Original, uncorrected Figs. 4 and 6 [file 41597_2026_6764_MOESM1_ESM.pdf]

Original, uncorrected figures

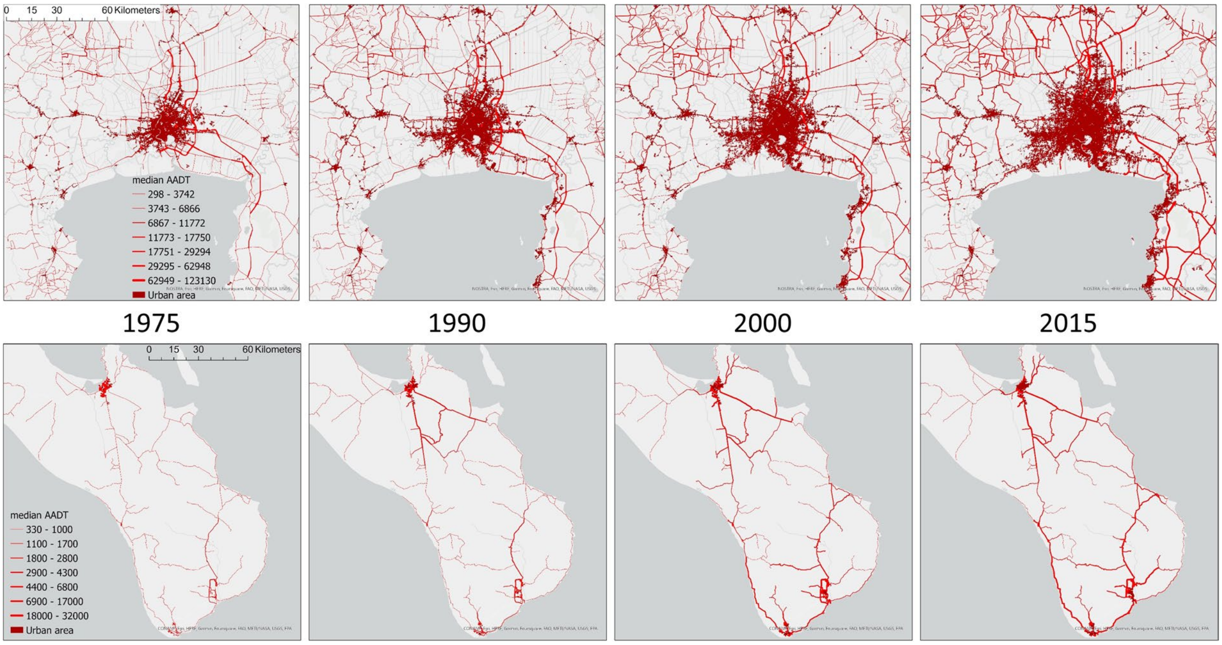

Fig. 4 Maps to illustrate the predictions of median Annual Average Daily Traffic (AADT) on extra-urban roads for the years 1975, 1990, 2000 and 2015. The top map series is for the region around the city of Bangkok in Thailand, whereas the bottom series depicts the southern tip of the Baja California Peninsula in Mexico (the main city in the maps is La Paz in the North). The expansion of the urban area as well as the growth in traffic volumes can be clearly seen in both map series.

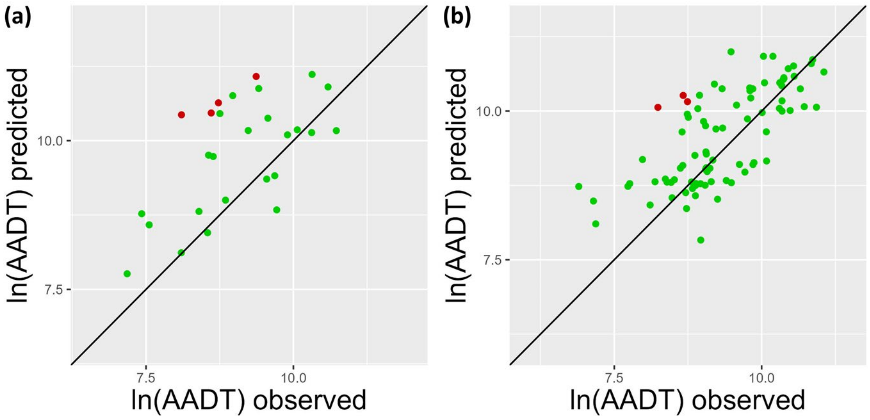

Fig. 6 Scatterplots of the 1975 observed and predicted ln(AADT) in (**a**) Switzerland and (**b**) the Netherlands. The black lines indicate perfect prediction (i.e. observed = predicted). The dot colours indicate whether an observed AADT is within (green) or outside (red) the 90% prediction interval. AADT = Annual Average Daily Traffic.
